# Effect of High-Intensity Interval Training versus Moderate-Intensity Continuous Training on Cardiorespiratory Fitness in Children and Adolescents: A Meta-Analysis

**DOI:** 10.3390/ijerph16091533

**Published:** 2019-04-30

**Authors:** Meng Cao, Minghui Quan, Jie Zhuang

**Affiliations:** 1 School of Physical Education and Sports Training, Shanghai University of Sport, Shanghai 200438, China; caomengsus@163.com; 2 School of Kinesiology, Shanghai University of Sport, Shanghai 200438, China; quanminghui@163.com

**Keywords:** high-intensity interval training, obesity, adolescent, cardiorespiratory fitness

## Abstract

Enhancing cardiorespiratory fitness (CRF) can lead to substantial health benefits. Comparisons between high-intensity interval training (HIIT) and moderate-intensity continuous training (MICT) on CRF for children and adolescents are inconsistent and inconclusive. The objective of this study was to perform a meta-analysis to compare the effects between HIIT and MICT on CRF in children and adolescents. We searched MEDLINE, PubMed, Web of Science, and Google Scholar to identify relevant articles. The standardized mean differences (SMD) and 95% confidence intervals (95% CI) were calculated to determine the pooled effect size of HIIT and MICT on CRF. A total of 563 subjects from 17 studies (18 effects) were identified. The pooled effect size was 0.51 (95% CI = 0.33–0.69) comparing HIIT to MICT. Moreover, intervention duration, exercise modality, work and rest ratio, and total bouts did not significantly modify the effect of HIIT on CRF. It is concluded that compared with endurance training, HIIT has greater improvements on cardiorespiratory fitness among children and adolescents.

## 1. Introduction

Cardiorespiratory fitness (CRF) is an objective reproducible physiological measure that reflects the functional influences of physical activity habits, genetics, and disease status [1]. The gold standard of CRF is considered as maximal oxygen uptake [2], which can be measured by using maximal graded cardiorespiratory test [3] or by using an indirect calculating method to estimate maximal oxygen uptake [4]. There is strong and consistent evidence from epidemiological studies that low CRF is associated with higher morbidity and mortality from all causes, including cardiovascular disease (CVD) and cancer [3]. Furthermore, higher levels of CRF in childhood and adolescence are associated with a healthier cardiovascular profile later in life [5]. However, the current status of CRF for children and adolescents is not optimistic, since a follow-up study reported that the CRF of 25.4 million people aged 6 to 19 years from 27 countries declined by 3.6% per decade from 1958 to 2003 [4]. Therefore, as the number of children and adolescents with low CRF gradually increases [6], effective interventions targeted at promoting the development of CRF in this population are particularly important.

High-intensity interval training (HIIT) refers to intermittent exercise that involves alternating short bursts of high-intensity activity with lower-intensity activity for recovery or rest [7,8]. Previous evidence indicated that traditional moderate-intensity continuous training (MICT) and HIIT can both increase CRF [9], which relate to benefits in CVD factors and all-cause mortality [10]. In recent years, a number of experimental studies compared the effects of HIIT and MICT on CRF in children and adolescents, but the findings were inconsistent and inconclusive. Some intervention studies demonstrated that HIIT intervention stimulated significant increases in relative CRF when compared with MICT [11,12,13,14], while other studies did not observe any difference between these two methods [15,16,17,18,19,20,21,22,23,24,25,26,27,28]. Although there have been some systematic review suggested that the effect of HIIT on CRF is better than MICT [29,30], they focused on obese populations [30], or compared effect sizes with a non-exercise control group rather than a MICT group [31]. Up to now, no quantitative review has been conducted to compare the effect of HIIT versus MICT on CRF in children and adolescents.

Therefore, our aim was to review existing evidence of the effects of HIIT versus MICT on improving CRF in healthy children and adolescents and identify potential moderators of intervention effects. The findings will provide theoretical reference and suggestions for future intervention strategies of CRF in children and adolescents.

## 2. Methods

### 2.1. Search Strategy

In line with the PRISMA (Preferred Reporting Items for Systematic Reviews and Meta-Analysis) Statement guidelines [32], a literature search was conducted for randomized controlled trials (RCTs) or controlled trials studying the effects of HIIT interventions on cardiorespiratory fitness. Electronic database searches were performed using PubMed, MEDLINE, Web of Science, and Google Scholar up to February 18 2019. Articles were searched by using the following search criteria: (high intensity interval OR high-intensity interval OR high intensity intermittent OR high-intensity intermittent OR sprint interval OR HIIT OR HIIE) AND (cardiorespiratory fitness) OR maximal oxygen uptake OR peak oxygen uptake OR VO2max OR CRF) AND (children [MeSH] OR adolescen* [MeSH] OR boy OR girl OR youth [MeSH] OR kids OR student*) AND English [lang]. 

The literature search, quality assessment, and data extraction were conducted independently by two authors (M.C. and M.Q.). Initially, studies that were clearly not relevant were removed before assessing all other titles and abstracts using pre-determined inclusion and exclusion criteria. Subsequently, the reviewers independently reviewed the full text of potentially eligible papers, such that each paper was reviewed in duplicate. Any disagreement between the reviewers for inclusion was resolved through group discussion (with the third reviewer J.Z.).

### 2.2. Eligibility Criteria and Selection

Studies were considered to be eligible for inclusion according to the following criteria: (1) Participants were untrained children and adolescents aged between 6 and 17 years; (2) Participants were healthy and not suffering from any kind of acute or chronic diseases; (3) Randomized or non-randomized controlled trials of ≥2 weeks [33]; (4) Included HIIT group and MICT group. MICT was classified as moderate-intensity as defined as an intensity that elicits a heart rate response of 55–69% ${\mathrm{HR}}_{\max}$ or 40–59% ${\dot{V}O}_{2\max}$ [7], HIIT intensity was classified as “all-out”, “maximal effort”, “≥90% ${\dot{V}O}_{2\mathrm{peak}}$” [34], “85–95% ${\mathrm{HR}}_{\max}$” [35] or “≥100% maximal aerobic speed (MAS) [36]; (5) The outcome measures for this meta-analysis were CRF related markers, such as ${\dot{V}O}_{2\max}$, ${\dot{V}O}_{2\mathrm{peak}}$, bouts of 20 meters shuttle run test (20mSRT), and Yo-Yo test distance; (6) Written in English. 

The exclusion criteria were as follows: (1) Uncontrolled and cross-sectional studies; (2) Performed on adults or animals; (3) Did not report the outcomes of CRF. Unpublished documents and grey literature like conference papers, dissertations, and patents were excluded as well. 

### 2.3. Data Extraction and Quality Assessment

Cochrane Consumers and Communication Review Group’s data extraction protocol [37] was used to extract participant information including sample size, age, sex, and weight status, characteristics of intervention (including exercise intensity, frequency, duration and modality), study design, and study outcomes. CRF data were extracted in the forms of pre- and post-training intervention means, and standard deviations (SDs). Dependent variables included ${\dot{V}O}_{2\max}$ or ${\dot{V}O}_{2\mathrm{peak}}$ reported in mL/kg/min or L/min (if relative values were not reported), bouts of 20mSRT, and Yo-Yo test distance. In studies that reported intermediate and post-intervention values, only final values for CRF were compared with baseline.

Two reviewers (M.C. and M.Q.) independently assessed the risk of bias of studies that met the inclusion criteria. Scoring discrepancies were resolved via consensus and inter-rater reliability was calculated using percentage agreement. Risk of bias for the 17 studies was assessed using an eight-item checklist adapted from the PRISMA statement [38]. A risk of bias score was awarded to each study based on an 8-point scale coded as clearly described (√), ‘absent’(×) or ‘unclear or inadequately described’(?), for each of the following criteria: (1) Eligibility criteria were specified; (2) Participants were randomly allocated to groups; (3) The groups were similar at baseline regarding the primary outcome(s); (4) There was blinding of all assessors who measured the primary outcome(s); (5) Data for primary outcome(s) were analyzed by ‘intention to treat’; (6) Dropout for primary outcome(s) was described, with <20% dropout of participants; (7) Conducted the sample size calculations and the study was adequately powered to detect changes in the primary outcome(s); and (8) Summary results for each group plus estimated effect size (difference between groups) and its precision (e.g., 95% CI) were reported. Criteria were added to create an overall risk of bias score: studies were graded as low risk if scoring 7–8, moderate risk if scoring 4–6, or high risk if scoring <4.

### 2.4. Data Analysis

A random effects meta-analysis was conducted to determine the pooled effect size of HIIT and MICT on CRF markers, using STATA (STATA 15.0, Stata Corp., College Station, TX, USA) to calculate the standardized mean difference (SMD). We performed analyses to determine the effect of the change in CRF for HIIT vs. MICT in each study. Distribution of effect size (ES) was determined to be heterogeneous if *Q* reached a significance level of *P* < 0.05 and the sampling error accounted for less than 75% of the observed variance [39]. Consistency (i.e., homogeneity) of effects was assessed using *I^2^*, whereby values of <25, 50, and 75 were considered to indicate low, moderate, and high heterogeneity, respectively [40]. As an explorative tool, publication bias was examined visually by funnel plot and the statistically by Egger’s test [41]. The Trim and Fill method was used to estimate the stability of the overall effect, and funnel plots were examined for asymmetry [42,43].

To test the robustness of our findings, sensitivity analysis was conducted by removing one primary included study each time from the meta-analysis. Subgroup analyses were conducted to examine the effect of modification based on their theoretical or empirical relation to changes in cardiorespiratory fitness, including intervention duration, exercise modality, work and rest ratio, and total bouts [33,44], and quality of included studies. 

## 3. Results

### 3.1. Search Result

**Figure 1:**
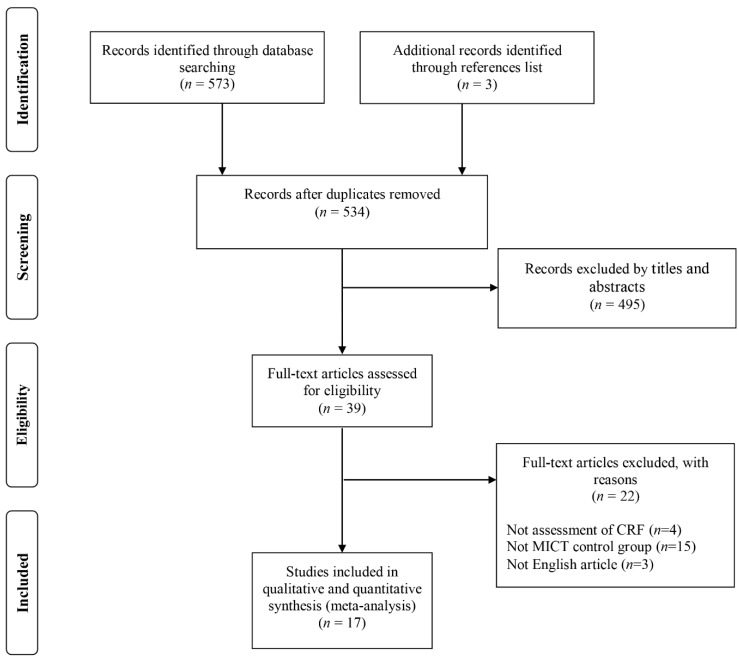
PRISMA (Preferred Reporting Items for Systematic Reviews and Meta Analyses) Flow diagram of the study selection process.

A search of electronic databases and a scan of article reference lists revealed 576 relevant studies and the screening process is shown in flow chart (Figure 1). Key study characteristics were extracted, including: country, size and source of study population, age, sex, weight status (normal weight, overweight or obesity), type of control group (endurance training), experimental group exercise mode, and intensity and length of intervention. Two effects were calculated and included from a study if the experimental design included a normal weight group and obese group, and the data for these two groups could not be combined [15]. After removal of duplicates and elimination of papers based on inclusion and exclusion criteria, a total of 17 studies were identified in this meta-analysis (Table 1).

### 3.2. Characteristics of Included Studies

Study characteristics are presented as mean ± SD unless otherwise stated. Eighteen effects from 17 RCTs [11,12,13,14,15,16,18,19,20,21,22,23,24,25,26,27,28] of 563 participants were included in the review (Table 1). Of the 17 included studies, 10 enrolled obese subjects [11,12,13,16,20,21,23,26,27,28], 1 included normal weight and obese subjects [39], and 6 included normal weight subjects [14,18,19,22,24,25]. Five studies included boys only [13,18,23,25,27], 2 studies did not reported gender [15,22] and the 10 remaining studies included both boys and girls [11,12,14,16,19,20,21,24,26,28]. Five studies enrolled children under 12 years old [14,18,19,24,25], and the other studies enrolled children aged 12 years old and older [11,12,13,15,16,20,21,22,23,26,27,28]. Sixteen studies were RCTs [11,12,13,15,16,18,19,20,21,22,23,24,25,26,27] and a 1 was a non-RCT [28]. Five studies were conducted in a school setting [14,18,19,22,24], and 12 studies were conducted in a clinical setting [11,12,13,15,16,20,21,23,25,26,27,28]. The duration of interventions of the included studies lasted from 3 weeks [23,25] to 24 weeks [16]. The intervention duration of included studies lasted 4 weeks [21], 5 weeks [14], 6 weeks [20], 8 weeks [15,18], 12 weeks [11,13,19,22,24,26,27,28], and 15 weeks [12]. Exercise training sessions were implemented twice a week in 3 studies [12,19,24] and 13 studies reported 3 sessions per week [11,13,14,16,18,20,21,22,23,25,26,27,28]. Kargarfard et al. reported 3 sessions per week in the HIIT group and 5 in the MICT group [15]. The mode of HIIT for interventions primarily involved running (11 studies) [12,13,14,15,16,19,22,23,24,27,28]; 5 studies administered a cycling protocol [11,18,20,21,26], and in one remaining study, exercise modality was not reported [25]. 

### 3.3. Risk of Bias

Methodological ‘risk of bias’ scores are provided in Table 2. Three studies were considered to have a high risk of bias [12,13,22], whereas thirteen [14,15,16,18,19,20,21,23,24,25,26,27,28] were moderate and one [11] was considered to have a low risk of bias.

### 3.4. Findings

**Figure 2:**
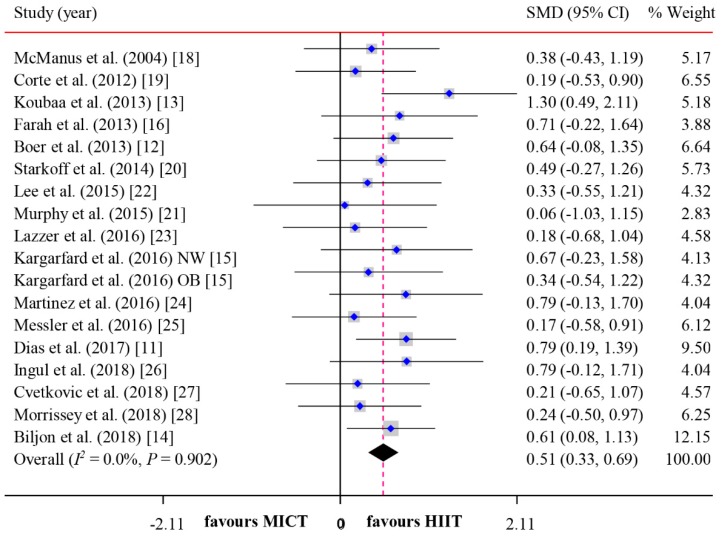
Forest plot for comparing the effects between HIIT and MICT interventions on CRF in children and adolescents by pooling 18 included studies.

**Figure 3:**
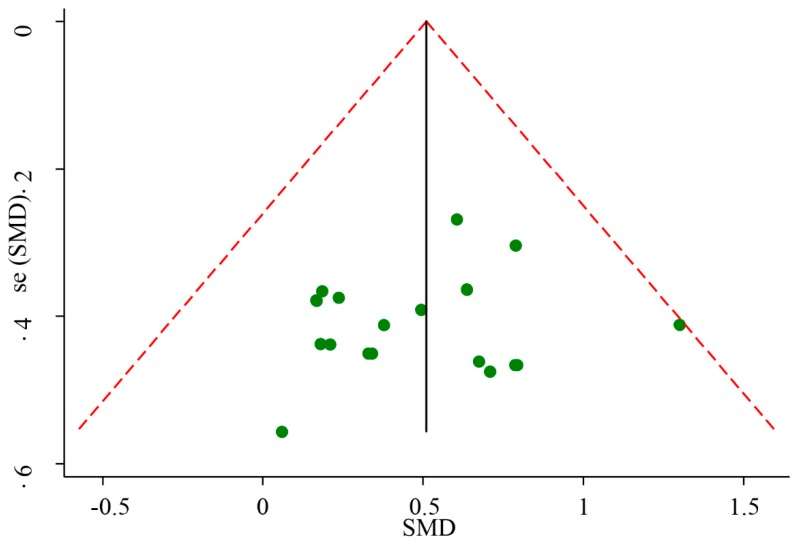
Funnel plot of standard difference in means versus standard error of 18 included studies.

**Figure 4:**
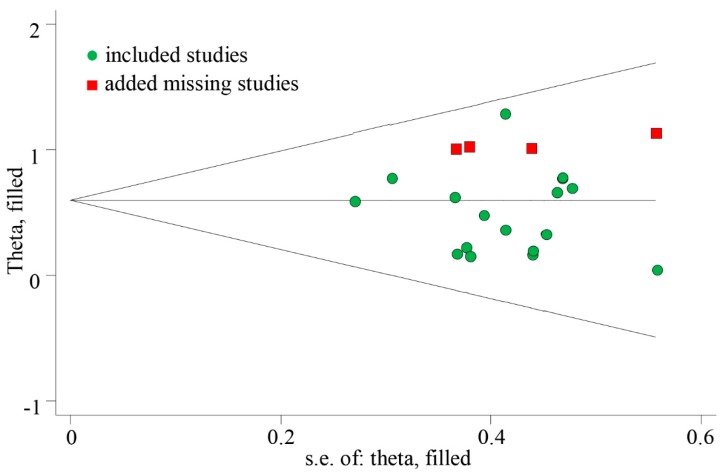
Results of trim and fill method (included 18 studies and 4 added studies).

The meta-analyzed effect of HIIT, when compared to MICT may have a moderate beneficial effect on CRF (SMD = 0.51, 95% CI = 0.33–0.69, *P* < 0.01) with high consistency of effects (*I^2^* = 0.00, *P* = 0.79) (Figure 2). Visual Egger’s test results showed no significant publication bias (P = 0.48), but the funnel plot exhibited asymmetry (Figure 3). Further analysis was undertaken using the trim and fill method to test the stability of the combined results. Four potential missing studies were added/filled; however, the random effect analysis showed no significant difference (0.51, 95% CI = 0.33–0.70 before filling versus 0.60, 95% CI = 0.43–0.76 after filling) and the combined results were still stable (Figure 4). Intervention duration (*P* = 0.34), exercise modality (*P* = 0.99), work and rest ratio (*P* = 0.26) and total bouts (*P* = 0.92) did not significant modify the effects of HIIT on CRF (Table 3).

## 4. Discussion

This meta-analysis aimed to directly compare effects of HIIT and MICT training protocols for improvement on cardiorespiratory fitness in children and adolescents. Our results revealed, firstly, that HIIT is more effective (SMD = 0.51, 95% CI = 0.33–0.69, *P* < 0.01) in improving CRF of children and adolescents aged 6 to 17 years when compared to MICT. Secondly, the overall effect was not significantly modified by intervention duration, exercise modality, ratio of work and rest, and total bouts. 

The findings of this study were consistent with a review that examined the effect of HIIT on fitness of obese children, which reported statistically significant effects for ${\dot{V}O}_{2\max}$ in their meta-analysis ranging from 1.6 to 3.7 mL·kg^−1^·min^−1^ [45]. Our results are similar to previous meta-analyses which have demonstrated that HIIT improves CRF with large effects in normal weight and overweight/obese adults [33]. A previous study showed that the improvement of CRF by 1 mL·kg^−1^·min^−1^, as assessed by a maximal bike test, reduced the risk for developing overweight or obesity by 10% in 6 years [46]. Therefore, in summary, we considered that HIIT shows promise as a time-efficient training method, yielding similar or greater improvements in CRF compared to MICT.

As noted in the subgroup analyses, the effects of HIIT were consistent, with average CRF improvements of 38–79% when compared with MICT, irrespective of modality, duration, work and rest ratio, total bouts, and risk of bias. The consistency of these results suggest that the findings of this meta-analysis are robust.

### 4.1. Potential Mechanism

Some of explanations might be given regarding why the effect of HIIT on CRF was better than MICT. First, mitochondrial adaptations to short-term training is a possible mechanism. In the study by MacInnis et al. [45], participants performed six training sessions with each leg over two weeks, with one leg performing HIIT and the other leg performing MICT, and the volume of training was identical for each leg. The results showed that HIIT compared to MICT elicited a greater increase in mitochondrial content, and HIIT induced greater increases in citrate synthase maximal activity, type Ⅱ fiber activation, adenosine monophosphate activated protein kinase activity [47] and mass-specific J_O2_ (oxygen flux) relative to MICT may be a contributing factor [48]. Second, previous studies indicated HIIT protocol was more effective on central adaptation, such as maximal stroke volume, cardiac output and blood volume, which are important components of CRF [49,50,51,52].

### 4.2. Strengths and Limitations

Some limitation also need to be considered. First, this review was not pre-registered on PROSPERO and we did not contact key experts in the field. Secondly, the evidence of change in CRF is largely limited by differences in measurement methods; CRF assessment methods varied across studies, which might generate heterogeneity and bias of overall effect estimation. However, the results of sensitivity analyses indicated that such differences are unlikely to affect the overall results. Thirdly, participants in each group performing different “doses” of exercise may another limitation. Fourthly, a publication bias was possible, caused by excluding studies published in other languages and grey literature (e.g., theses, dissertations), though statistical tests do not suggest a publication bias in the present meta-analysis. Finally, this meta-analysis has combined the findings from the most comprehensive and up-to-date literature.

## 5. Conclusions

This meta-analysis review indicates that HIIT is a better training methodology to improve cardiorespiratory fitness among healthy children and adolescents compared to MICT. Considering its characteristics of effectiveness and efficiency, HIIT may be an effective approach to achieve improvements in CRF among healthy children and adolescents. Future studies also need to further analyze the effect of HIIT on other domains of physical fitness (e.g., sprint capacity, running performance and countermovement jump, etc.), in order to enhance its efficiency on health-related outcomes among children and adolescents.

## Figures and Tables

**Table 1:** Characteristics of 18 included studies examining the effect of high-intensity interval training on cardiorespiratory fitness.

Study	Year	Sample Population	Duration (week)	Group (*n*)	Modality/ Intensity	Repeated Bouts/ Frequency	Work/rest W/R Ratio	Exercise Time per Week (min)	CRF Outcomes
McManus et al. [18] Country: China Study design: RCT	2004	school children; *n* = 25 (25 boys; 10.3 ± 0.5 years)	8	HIIT (10) MICT (15)	Cycling (all-out) Cycling (85% HR_max_)	Bouts: 7 (3 times weekly)	30 s/ 165 s 0.18	10.5 60	${\dot{V}O}_{2\max}$ (mL·kg^−1^·min^−1^)
Corte et al. [19] Country: Brazil Study design: RCT	2012	school children; *n* = 30 (9 boys; 10.4 ± 0.9 years)	12	HIIT (15) MICT (15)	Running (100% ${\dot{V}O}_{2\mathrm{peak}}$) Running (80% HR_peak_)	Bouts: 3–6 (2 times weekly)	60 s/ 180 s 0.33	6–18 60–120	${\dot{V}O}_{2\max}$ (mL·kg^−1^·min^−1^)
Koubaa et al. [13] Country: Tunisia Study design: RCT	2013	obese children; *n* = 29 (29 boys; 13.0 ± 0.8 years)	12	HIIT (14) MICT (15)	Running (80–90% ${\dot{V}O}_{2\max}$) Running (60–70% ${\dot{V}O}_{2\max}$)	Bouts: NR (3 times weekly)	120 s/ 60 s 2.00	NA NA	${\dot{V}O}_{2\max}$ (mL·kg^−1^·min^−1^)
Farah et al. [16] Country: Brazil Study design: RCT	2013	obese children; *n* = 19 (9 boys; 15.1 ± 1.2 years)	24	HIIT (9) MICT (10)	Running (100% VT) Running (80% VT)	Bouts: NR (3 times weekly)	NA	NA NA	${\dot{V}O}_{2\max}$ (mL·kg^−1^·min^−1^)
Boer et al. [12] Country: Blegium Study design: RCT	2013	obese children; *n* = 32 (9 boys; 17.0 ± 3.0 years)	15	HIIT (17) MICT (15)	Running (110% VT) Running (100% VT)	Bouts: 10 (2 times weekly)	15 s/ 45 s 0.33	5 60	${\dot{V}O}_{2\max}$ (mL·kg^−1^·min^−1^)
Starkoff et al. [20] Country: USA Study design: RCT	2014	obese children; *n* = 27 (10 boys; 14.7 ± 1.5 years)	6	HIIT (14) MICT (13)	Cycling (90–95% APMHR) Cycling (65–70% APMHR)	Bouts: 10 (3 times weekly)	120 s/ 60 s 2.00	60 90	${\dot{V}O}_{2\max}$ (mL·kg^−1^·min^−1^)
Lee et al. [22] Country: Korea Study design: RCT	2015	school children; *n* = 20 (NR; 15.3 ± 2.2 years)	12	HIIT (10) MICT (10)	Running (≥80% HRR) Running (<40% HRR)	Bouts: NR (3 times weekly)	30 s/ 30 s 1.00	NA NA	${\dot{V}O}_{2\max}$ (mL·kg^−1^·min^−1^)
Murphy et al. [21] Country: USA Study design: RCT	2015	obese children; *n* = 13 (3 boys; 13.7 ± 2.0 years)	4	HIIT (6) MICT (7)	Cycling (80–90% HR_max_) Cycling (65% HR_max_)	Bouts: 10 (3 times weekly)	60 s/ 120 s 0.50	30 90	${\dot{V}O}_{2\max}$ (mL·kg^−1^·min^−1^)
Lazzer et al. [23] Country: Italy Study design: RCT	2016	obese children; *n* = 30 (30 boys; 13.7 ± 2.0 years)	3	HIIT (10) MICT (20)	Running (100% ${\dot{V}O}_{2\max}$) Running (70% ${\dot{V}O}_{2\max}$)	Bouts: 6 (3 times weekly)	40 s/ 300 s 0.13	12 120	${\dot{V}O}_{2\max}$ (mL·kg^−1^·min^−1^)
Kargarfard et al.-NW [15] Country: Iran Study design: RCT	2016	school children; *n* = 20 (NR; 12.2 ± 1.5 years)	8	HIIT (10) MICT (10)	Running (60–90% HRR) Running (60–70% HRR)	Bouts: 8-10 (3 times weekly)	240 s/ 120 s 2.00	96–120 150–180	${\dot{V}O}_{2\max}$ (mL·kg^−1^·min^−1^)
Kargarfard et al.-OB [15] Country: Iran Study design: RCT	2016	obese children; *n* = 20 (NR; 12.3 ± 1.3 years)	8	HIIT (10) MICT (10)	Running (60–90% HRR) Running (60–70% HRR)	Bouts: 8–10 (3 times weekly)	240 s/ 120 s 2.00	96–120 150–180	${\dot{V}O}_{2\max}$ (mL·kg^−1^·min^−1^)
Martinez et al. [24] Country: Spain Study design: RCT	2016	school children; *n* = 94 (52 boys; 8.2 ± 0.7 years)	12	HIIT (38) MICT (56)	Running and jumping (NR) Aerobic exercise (NR)	Bouts: NR (2 times weekly)	10–20 s / NR NA	40 40	${\dot{V}O}_{2\max}$ (mL·kg^−1^·min^−1^)
Messler et al. [25] Country: Germany Study design: RCT	2016	ADHD adolescents *n* = 28 (28 boys; 11.0 ± 1.0 years)	3	HIIT (14) MICT (14)	NR (95% HR_peak_) Mixture (<70% HR_peak_)	Bouts: 4 (3 times weekly)	240 s/ 180 s 1.33	48 180	${\dot{V}O}_{2\max}$ (L·min^−1^)
Dias et al. [11] Country: Australia Study design: RCT	2017	obese children; *n* = 47 (31 boys; 12.2 ± 2.1 years)	12	HIIT (25) MICT (22)	Cycling (85–95% HR_max_) Cycling (60–70% HR_max_)	Bouts: 4 (3 times weekly)	240 s/ 180 s 1.33	48 132	${\dot{V}O}_{2\max}$ (mL·kg^−1^·min^−1^)
Ingul et al. [26] Country: Norway Study design: RCT	2018	obese children; *n* = 41 (21 boys; 12.0 ± 2.3 years)	12	HIIT (17) MICT (24)	Cycling (85–95% HR_max_) Cycling (60–70% HR_max_)	Bouts: 4 (3 times weekly)	240 s/ 180 s 1.33	48 132	${\dot{V}O}_{2\max}$ (mL·kg^−1^·min^−1^)
Cvetkovic et al. [27] Country: Serbia Study design: RCT	2018	obese children; *n* = 21 (21 boys; 11–13 years)	12	HIIT (11) MICT (10)	Running (100% MAS) Running (NR)	Bouts: 5–10 (3 times weekly)	10–20 s / 10–20 s 1.00	7.5–15 180	Yo−Yo test distance
Morrissey et al. [28] Country: France Study design: non-RCT	2018	obese children; *n* = 29 (8 boys; 15.2±1.4 years)	12	HIIT (16) MICT (13)	Running (90–95 % HR_max_) Running (60–70 % HR_max_)	Bouts: 4–6 (3 times weekly)	120–150 s/ 90 s 1.33–1.66	24–45 120–180	20mSRT bouts
Biljon et al. [14] Country: South Africa Study design: RCT	2018	school children; *n* = 58 (26 boys; 11.1 ± 0.8 years)	5	HIIT (29) MICT (29)	Running (>80% HR_max_) Running (65–70% HR_max_)	Bouts: 10 (3 times weekly)	60 s/ 75 s 0.80	30 99	${\dot{V}O}_{2\max}$ (mL·kg^−1^·min^−1^)

APMHR, age predict maximal heart rate; CRF, cardiorespiratory fitness; HIIT, high-intensity interval training; HR_peak_, peak heart rate; HR_max_, maximal heart rate; HRR, heart rate reserve; MAS, maximal aerobic speed; MICT, moderate-intensity continuous training; NA, not applicable; NC, no changes P > 0.05; NR, not report; P_peak_, peak power; V_peak_, peak velocity; VO_2max_, maximal oxygen uptake; VT, ventilatory threshold.

**Table 2:** Quality assessment / risk of bias of 18 included studies.

Study	Year	*n*	Age	1	2	3	4	5	6	7	8	Total	Risk of Bias
McManus et al. [18]	2004	25	10.3 ± 0.6	√	×	√	×	×	√	?	√	4	Medium risk
Corte et al. [19]	2012	30	10.4 ± 0.9	√	√	√	×	?	√	?	√	5	Medium risk
Koubaa et al. [13]	2013	29	13.0 ± 0.8	×	×	×	×	×	×	?	×	0	High risk
Farah et al. [16]	2013	19	15.1 ± 1.2	√	√	√	√	×	×	?	?	4	Medium risk
Boer et al. [12]	2013	32	17.0 ± 3.0	√	×	?	×	×	√	?	?	2	High risk
Starkoff et al. [20]	2014	27	14.7 ± 1.5	√	√	√	×	×	√	√	√	6	Medium risk
Lee et al. [22]	2015	20	15.3 ± 2.2	√	√	√	×	?	?	×	×	3	High risk
Murphy et al. [21]	2015	13	13.7 ± 2.0	√	?	√	×	×	√	?	√	4	Medium risk
Lazzer et al. [23]	2016	30	16.8 ± 0.7	√	√	√	×	√	√	×	√	6	Medium risk
Kargarfard et al. [15]	2016	20	12.4 ± 1.3	√	√	√	×	√	√	×	√	6	Medium risk
Martinez et al. [24]	2016	94	8.2 ± 0.7	√	√	√	×	√	×	?	√	5	Medium risk
Messler et al. [25]	2016	28	11.0 ± 1.0	√	√	√	×	√	?	?	√	5	Medium risk
Dias et al. [11]	2017	47	12.2 ± 2.1	√	√	√	×	√	√	√	√	7	Low risk
Ingul et al. [26]	2018	41	12.0 ± 3.3	√	√	√	×	√	√	×	√	6	Medium risk
Cvetkovic et al. [27]	2018	21	11.0–13.0	√	√	√	×	√	√	?	√	6	Medium risk
Morissey et al. [28]	2018	29	15.2 ± 1.4	√	×	√	×	√	√	√	√	6	Medium risk
Biljon et al. [14]	2018	58	11.1 ± 0.8	√	×	√	×	√	√	√	√	6	Medium risk

Criteria: (1) Eligibility criteria were specified; (2) Participants were randomly allocated to groups; (3) The groups were similar at baseline regarding the primary outcome(s); (4) There was blinding of all assessors who measured the primary outcome(s); (5) Data for primary outcome(s) were analyzed by ‘intention to treat’; (6) Dropout for primary outcome(s) was described, with <20% dropout of participants; (7) Conducted the sample size calculations and the study was adequately powered to detect changes in the primary outcome(s); and (8) Summary results for each group plus estimated effect size (difference between groups) and its precision (e.g., 95% CI) were reported. Coding: ‘clearly described’ (√), ‘absent’ (×) or ‘unclear or inadequately described’ (?).

**Table 3:** Subgroup analysis of the effects comparison of HIIT and MICT interventions on CRF in children and adolescents.

Subgroup	No. of Trials/Total No. (%)	Subjects (*n*)	SMD (95% CI)	Favors HIIT	Favors MICT	*P* Value	*P* for Interaction
All studies	18/18 (100)	563	0.51 (0.33, 0.69)	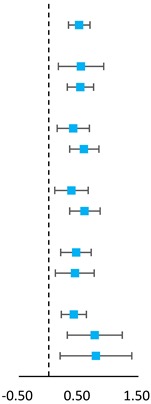		0.00	
Modality of INT							
Cycling	5/17 (29)	153	0.54 (0.16, 0.92)			0.04	0.94
Running	12/17 (71)	382	0.53 (0.31, 0.75)			0.00	
INT duration							
≤8 weeks	8/18 (44)	212	0.41 (0.14, 0.68)			0.01	0.23
>8 weeks	10/18 (56)	351	0.59 (0.35, 0.84)			0.00	
Work: rest ratio							
≤1	8/17 (47)	207	0.38 (0.10, 0.66)			0.08	0.16
>1	9/17 (53)	252	0.60 (0.35, 0.86)			0.00	
Total bouts							
≤180	8/14 (57)	242	0.46 (0.20, 0.71)			0.01	0.91
>180	6/14 (43)	149	0.44 (0.11, 0.76)			0.01	
Risk of bias							
high	3/18 (17)	41	0.42 (0.21, 0.63)			0.00	0.75
Medium	14/18 (77)	497	0.77 (0.19, 1.23)			0.00	
Low	1/18 (6)	25	0.79 (0.33, 1.39)			0.01	

CRF, Cardiorespiratory fitness; HIIT, high-intensity interval training; MICT, moderate-intensity continuously training; INT, intervention.

## References

[1] Sui X., Lamonte M.J., Blair S.N. (2007). Cardiorespiratory fitness as a predictor of nonfatal cardiovascular events in asymptomatic women and men. Am. J. Epidemiol..

[2] Ross R., Blair S.N., Arena R., Church T.S., Després J.P., Franklin B.A., Haskell W.L., Kaminsky L.A., Levine B.D., Lavie C.J. (2016). Importance of assessing cardiorespiratory fitness in clinical practice: A case for fitness as a clinical vital sign: A scientific statement from the american heart association. Circulation.

[3] Kodama S., Saito K., Tanaka S., Maki M., Yachi Y., Asumi M., Sugawara A., Totsuka K., Shimano H., Ohashi Y. (2009). Cardiorespiratory fitness as a quantitative predictor of all-cause mortality and cardiovascular events in healthy men and women: A meta-analysis. J. Am. Med. Assoc..

[4] Tomkinson G.R., Olds T.S. (2007). Secular changes in pediatric aerobic fitness test performance: The global picture. Med. Sport Sci..

[5] Ruiz J.R., Castro-Piñero J., Artero E.G., Ortega F.B., Sjöström M., Suni J., Castillo M.J. (2009). Predictive validity of health-related fitness in youth: A systematic review. Br. J. Sports Med..

[6] Martinez-Gomez D., Ortega F.B., Ruiz J.R., Vicente-Rodriguez G., Veiga O.L., Widhalm K., Manios Y., Béghin L., Valtueña J., Kafatos A. (2011). Excessive sedentary time and low cardiorespiratory fitness in European adolescents: The HELENA study. Arch. Dis. Childh..

[7] Norton K., Norton L., Sadgrove D. (2010). Position statement on physical activity and exercise intensity terminology. J. Sci. Med. Sport.

[8] Sloth M., Sloth D., Overgaard K., Dalgas U. (2013). Effects of sprint interval training on VO_2max_ and aerobic exercise performance: A systematic review and meta-analysis. Scand. J. Med. Sci. Sports.

[9] Hottenrott K., Ludyga S., Schulze S. (2012). Effects of high intensity training and continuous endurance training on aerobic capacity and body composition in recreationally active runners. J. Sports Sci. Med..

[10] Oja P., Titze S., Bauman A., De Geus B., Krenn P., Reger-Nash B., Kohlberger T. (2011). Health benefits of cycling: A systematic review. Scand. J. Med. Sci. Sports.

[11] Dias K.A., Ingul C.B., Tjønna A.E., Keating S.E., Gomersall S.R., Follestad T., Hosseini M.S., Hollekim-Strand S.M., Ro T.B., Haram M. (2018). Effect of high-intensity interval training on fitness, fat mass and cardiometabolic biomarkers in children with obesity: A randomised controlled trial. Sports Med..

[12] Boer P.H., Meeus M., Terblanche E., Rombaut L., Wandele I.D., Hermans L., Gysel T., Ruige J., Calders P. (2014). The influence of sprint interval training on body composition, physical and metabolic fitness in adolescents and young adults with intellectual disability: A randomized controlled trial. Clin. Rehabil..

[13] Koubaa A. (2013). Effect of intermittent and continuous training on body composition cardiorespiratory fitness and lipid profile in obese adolescents. IOSR J. Pharm..

[14] Van Biljon A., McKune A.J., DuBose K.D., Kolanisi U., Semple S.J. (2018). Do short-term exercise interventions improve cardiometabolic risk factors in children?. J. Pediatr..

[15] Kargarfard M., Lam E.T., Shariat A., Asle Mohammadi M., Afrasiabi S., Shaw I., Shaw B.S. (2016). Effects of endurance and high intensity training on ICAM-1 and VCAM-1 levels and arterial pressure in obese and normal weight adolescents. Phys. Sportsmed..

[16] Farah B.Q., Ritti-Dias R.M., Balagopal P.B., Hill J.O., Prado W.L. (2014). Does exercise intensity affect blood pressure and heart rate in obese adolescents? A 6-month multidisciplinary randomized intervention study. Pediatr. Obes..

[17] Berger N.J., Tolfrey K., Williams A.G., Jones A.M. (2007). Influence of continuous and interval training on oxygen uptake on-kinetics. Med. Sci. Sports Exerc..

[18] Mcmanus A.M., Cheng C.H., Leung M.P., Yung T.C., Macfarlane D.J. (2005). Improving aerobic power in primary school boys: A comparison of continuous and interval training. Int. J. Sports Med..

[19] De Araujo A.C.C., Roschel H., Picanço A.R., do Prado D.M.L., Villares S.M.F., de Sá Pinto A.L., Gualano B. (2012). Similar health benefits of endurance and high-intensity interval training in obese children. PLoS ONE.

[20] Starkoff B.E., Eneli I.U., Bonny A.E., Hoffman R.P., Devor S.T. (2014). Estimated aerobic capacity changes in adolescents with obesity following high intensity interval exercise. Int. J. Kinesiol. Sport Sci..

[21] Murphy A., Kist C., Gier A.J., Edwards N.M., Gao Z., Siegel R.M. (2015). The feasibility of high-intensity interval exercise in obese adolescents. Clin. Pediatr..

[22] Soo L.S., Ho Y.J., Seok S.Y. (2015). Effect of the low- versus high-intensity exercise training on endoplasmic reticulum stress and GLP-1 in adolescents with type 2 diabetes mellitus. J. Phys. Ther. Sci..

[23] Lazzer S., Tringali G., Caccavale M., De Micheli R., Abbruzzese L., Sartorio A. (2016). Effects of high-intensity interval training on physical capacities and substrate oxidation rate in obese adolescents. J. Endocrinol. Investig..

[24] Martínez S.R., Ríos L.J.C., Tamayo I.M., Almeida L.G., López-Gomez M.A., Jara C.C. (2016). An after-school, high-intensity, interval physical activity programme improves health-related fitness in children. Motriz. Rev. Educ. Fis..

[25] Meßler C.F., Holmberg H.C., Sperlich B. (2016). Multimodal therapy involving high-intensity interval training improves the physical fitness, motor skills, social behavior, and quality of life of boys with adhd: A randomized controlled study. J. Atten. Dis..

[26] Ingul C.B., Dias K.A., Tjonna A.E., Follestad T., Hosseini M.S., Timilsina A.S., Hollekim-Strand S.M., Ro T.B., Davies P.S., Cain P.A. (2018). Effect of high intensity interval training on cardiac function in children with obesity: A randomised controlled trial. Progr. Cardiovasc. Dis..

[27] Cvetković N., Stojanović E., Stojiljković N., Nikolić D., Scanlan A.T., Milanović Z. (2018). Exercise training in overweight and obese children: Recreational football and high-intensity interval training provide similar benefits to physical fitness. Scand. J. Med. Sci. Sports.

[28] Morrissey C., Montero D., Raverdy C., Masson D., Amiot M.J., Vinet A. (2018). Effects of exercise intensity on microvascular function in obese adolescents. Int. J. Sports Med..

[29] Thivel D., Masurier J., Baquet G., Timmons B.W., Pereira B., Berthoin S., Duclos M., Aucouturier J. (2018). High-intensity interval training in overweight and obese children and adolescents: Systematic review and meta-analysis. J. Sports Med. Phys. Fit..

[30] García-Hermoso A., Cerrillo-Urbina A.J., Herrera-Valenzuela T., Cristi-Montero C., Saavedra J.M., Martínez-Vizcaíno V. (2016). Is high-intensity interval training more effective on improving cardiometabolic risk and aerobic capacity than other forms of exercise in overweight and obese youth? A meta-analysis. Obes. Rev..

[31] Costigan S.A., Eather N., Plotnikoff R.C., Taaffe D.R., Lubans D.R. (2015). High-intensity interval training for improving health-related fitness in adolescents: A systematic review and meta-analysis. Br. J. Sports Med..

[32] Liberati A., Altman D.G., Tetzlaff J., Mulrow C., Gøtzsche P.C., Ioannidis J.P., Clarke M., Devereaux P.J., Kleijnen J., Moher D. (2009). The PRISMA statement for reporting systematic reviews and meta-analyses of studies that evaluate healthcare interventions: Explanation and elaboration. Epidemiol. Biostat. Pub. Health.

[33] Batacan R.B., Duncan M.J., Dalbo V.J., Tucker P.S., Fenning A.S. (2017). Effects of high-intensity interval training on cardiometabolic health: A systematic review and meta-analysis of intervention studies. Br. J. Sports Med..

[34] Gibala M.J., Mcgee S.L. (2008). Metabolic adaptations to short-term high-intensity interval training: A little pain for a lot of gain?. Exerc. Sport Sci. Rev..

[35] Weston K.S., Wisloff U., Coombes J.S. (2014). High-intensity interval training in patients with lifestyle-induced cardiometabolic disease: A systematic review and meta-analysis. Br. J. Sports Med..

[36] Baquet G., Gamelin F.X., Mucci P., Thévenet D., Van Praagh E., Berthoin S. (2010). Continuous vs. interval aerobic training in 8- to 11-year-old children. J. Strength Cond. Res..

[37] Lloyd Jones M.C., Morris M.G., Jakeman J.R. (2017). Impact of time and work:rest ratio matched sprint interval training programmes on performance: A randomised controlled trial. J. Sci. Med. Sport.

[38] Buchheit M., Laursen P.B. (2013). High-intensity interval training, solutions to the programming puzzle: Part I: Cardiopulmonary emphasis. Sports Med..

[39] Hedges L.V., Olkin I. (1985). Statistical Methods for Meta-Analysis.

[40] Higgins J.P., Thompson S.G., Deeks J.J., Altman D.G. (2003). Measuring inconsistency in meta-analyses. Br. Med. J..

[41] Egger M., Smith G.D., Schneider M., Minder C. (1997). Bias in meta-analysis detected by a simple, graphical test. BMJ Br. Med. J..

[42] Duval S., Tweedie R. (2000). Trim and fill: A simple funnel-plot-based method of testing and adjusting for publication bias in meta-analysis. Biometrics.

[43] Weinhandl E.D., Duval S. (2012). Generalization of trim and fill for application in meta-regression. Res. Synth. Methods.

[44] Dobbins M., Husson H., DeCorby K., LaRocca R.L. (2009). School-based physical activity programs for promoting physical activity and fitness in children and adolescents aged 6–18. Cochrane Database Syst. Rev..

[45] MacInnis M.J., Zacharewicz E., Martin B.J., Haikalis M.E., Skelly L.E., Tarnopolsky M.A., Murphy R.M., Gibala M.J. (2017). Superior mitochondrial adaptations in human skeletal muscle after interval compared to continuous single-leg cycling matched for total work. J. Physiol..

[46] Ortega F.B., Labayen I., Ruiz J.R., Kurvinen E., Loit H.M., Harro J., Veidebaum T., Sjöström M. (2011). Improvements in fitness reduce the risk of becoming overweight across puberty. Med. Sci. Sports Exerc..

[47] Kristensen D.E., Albers P.H., Prats C., Baba O., Birk J.B., Wojtaszewski J.F. (2015). Human muscle fibre type-specific regulation of AMPK and downstream targets by exercise. J. Physiol..

[48] Larsen S., Nielsen J., Hansen C.N., Nielsen L.B., Wibrand F., Stride N., Schroder H.D., Boushel R., Helge J.W., Dela F. (2012). Biomarkers of mitochondrial content in skeletal muscle of healthy young human subjects. J. Physiol..

[49] Astorino T.A., Allen R.P., Roberson D.W., Jurancich M. (2012). Effect of high-intensity interval training on cardiovascular function, VO_2max_, and muscular force. J. Strength Condit. Res..

[50] Helgerud J., Høydal K., Wang E., Karlsen T., Berg P., Bjerkaas M., Simonsen T., Helgesen C., Hjorth N., Bach R. (2007). Aerobic high-intensity intervals improve VO_2max_ more than moderate training. Med. Sci. Sports Exerc..

[51] Daussin F.N., Zoll J., Dufour S.P., Ponsot E., Lonsdorfer-Wolf E., Doutreleau S., Mettauer B., Piquard F., Geny B., Richard R. (2008). Effect of interval versus continuous training on cardiorespiratory and mitochondrial functions: Relationship to aerobic performance improvements in sedentary subjects. Am. J. Physiol..

[52] Baekkerud F.H., Solberg F., Leinan I.M., Wisløff U., Karlsen T., Rognmo Ø. (2016). Comparison of three popular exercise modalities on VO_2max_ in overweight and obese. Med. Sci. Sports Exerc..

